# A negative feedback loop between KLF9 and the EMT program dictates metastasis of hepatocellular carcinoma

**DOI:** 10.1111/jcmm.17823

**Published:** 2023-07-03

**Authors:** Tao Wang, Limin Feng, Zhong Shi, Lixian Yang, Xiaofu Yu, Jinsong Wu, Jirui Sun, Jinku Zhang, Yuxiong Feng, Weilin Wang

**Affiliations:** ^1^ Department of Interventional Oncology Renji Hospital, Shanghai Jiao Tong University School of Medicine Shanghai China; ^2^ Zhejiang University School of Medicine Hangzhou China; ^3^ Department of Medical Oncology Zhejiang Cancer Hospital, Hangzhou Institute of Medicine (HIM), Chinese Academy of Sciences Hangzhou China; ^4^ First Affiliated Hospital Institute of Translational Medicine, Zhejiang University School of Medicine Hangzhou China; ^5^ Cancer Center Zhejiang University Hangzhou China; ^6^ Department of Thoracic Radiotherapy Zhejiang Cancer Hospital, Hangzhou Institute of Medicine (HIM), Chinese Academy of Sciences Hangzhou China; ^7^ College of Biomedical Engineering and Instrument Science Zhejiang University Hangzhou China; ^8^ Department of Pathology Baoding NO.1 Central Hospital Baoding China; ^9^ Department of Hepatobiliary and Pancreatic Surgery The Second Affiliated Hospital, Zhejiang University School of Medicine Hangzhou China; ^10^ Key Laboratory of Precision Diagnosis and Treatment for Hepatobiliary and Pancreatic Tumor of Zhejiang Province Hangzhou China; ^11^ Research Center of Diagnosis and Treatment Technology for Hepatocellular Carcinoma of Zhejiang Province Hangzhou China

**Keywords:** epithelial–mesenchymal transition, hepatocellular carcinoma, KLF9, metastasis, slug

## Abstract

Metastasis is the primary cause of death of hepatocellular carcinoma (HCC), while the mechanism underlying this severe disease remains largely unclear. The Kruppel‐like factor (KLF) family is one of the largest transcription factor families that control multiple physiologic and pathologic processes by governing the cellular transcriptome. To identify metastatic regulators of HCC, we conducted gene expression profiling on the MHCC97 cell series, a set of subclones of the original MHCC97 that was established by in vivo metastasis selection therefore harbouring differential metastatic capacities. We found that the expression of KLF9, a member of the KLF family, was dramatically repressed in the metastatic progeny clone of the MHCC97 cells. Functional studies revealed overexpression of KLF9 suppressed HCC migration in vitro and metastasis in vivo, while knockdown of KLF9 was sufficient to promote cell migration and metastasis accordingly. Mechanistically, we found the expression of KLF9 can reverse the pro‐metastatic epithelial‐mesenchymal transition (EMT) program via direct binding to the promoter regions of essential mesenchymal genes, thus repressing their expression. Interestingly, we further revealed that KLF9 was, in turn, directly suppressed by a mesenchymal transcription factor Slug, suggesting an intriguing negative feedback loop between KLF9 and the EMT program. Using clinical samples, we found that KLF9 was not only downregulated in HCC tissue compared to its normal counterparts but also further reduced in the HCC samples of whom had developed metastatic lesions. Together, we established a critical transcription factor that represses HCC metastasis, which is clinically and mechanically significant in HCC therapies.

## 1 INTRODUCTION

Hepatocellular carcinoma (HCC) is the sixth most common cancer worldwide, with approximately 1 million new cases worldwide and about 0.8 million deaths in 2020. The high mortality of HCC makes it the third leading cause of cancer‐related death.^1^ The major risk factors for HCC include chronic infection with hepatitis B virus (HBV) or hepatitis C virus (HCV), aflatoxin contamination, alcohol consumption, obesity, diabetes and nonalcoholic fatty liver disease (NAFLD).^2^

Like other types of cancers, the major cause of HCC‐related death is metastasis. Due to its intrinsic biological features, HCC usually progresses swiftly and is prone to develop post‐therapeutic recurrence and metastasis. The most frequent metastatic sites of HCC are the liver and lung, where the metastatic lesions could readily turn into life‐threatening complications.^3^ Therefore, revealing the underlying mechanism of HCC metastasis is of substantial significance, which is the key to the discovery of targeted therapy against this deadly disease. Epithelial–mesenchymal transition (EMT) is a multistep developmental program that could endow cancer cells with metastatic capacity. Many reports have shown that activation of the EMT programme in cancer cells contributes to metastasis by enhancing cell mobility, invasion and resistance to apoptosis in many types of cancers, HCC included.^4^ On the contrary, a reversal of this program, namely mesenchymal–epithelial transition (MET), can repress the malignant phenotypes caused by EMT. Thus, identifying potential EMT suppressors, or inducers of MET, can provide therapeutic targets for controlling HCC metastasis.

The Krüppel‐like factor (KLF) family is one of the largest protein families of zinc finger transcription factors that regulate several cellular processes, including proliferation, differentiation and migration.^5^ By directly setting the landscape of cellular transcriptome, the KLF family members have been found to participate in the initiation and progression of many cancers, and several members have been reported to function in HCC at present.^6^ However, while they have been implicated in the process of cell proliferation and survival of HCC, very little is known if the KLF family proteins can directly govern the metastatic potential of HCC. Interestingly, some members of the KLF family, in particular KLF4, are essential drivers of cell fate determination and differentiation.^7^, ^8^ Beyond its function in cancer, EMT, or MET, is a molecular program governing cell state transition. Therefore, it will be interesting to identify potential members of the KLF family that might regulate the dynamics of EMT/MET to control metastasis of HCC.

Using a series of progeny clones derived from the same ancestor HCC primary cell but displaying different metastatic potentials, we aimed to identify KLF family members that could regulate HCC metastasis. According to the results of an unbiased transcriptome profiling, we found that the expression of KLF9 was strongly negatively correlated with the metastatic potential of HCC cells. Furthermore, functional studies revealed that KLF9 was a potent repressor of HCC metastasis. Interestingly, the anti‐metastatic function of KLF9 in HCC was likely due to its role in suppressing the activation of EMT, while KLF9 was, in turn, repressed by Slug, an important factor of the EMT programme, suggesting a negative feedback loop between KLF9 and the EMT program. Taking together, we identified an essential transcription factor of the KLF family that can repress liver cancer metastasis by controlling the epithelial–mesenchymal dynamics of HCC cells.

## 2 MATERIALS AND METHODS

### 2.1 HCC tissue samples and cell lines

Forty‐three pairs of primary HCC and their matched adjacent normal liver tissues, which were at least 1.5–2.0 cm away from cancer, were collected from HCC patients treated at the Renji Hospital, Shanghai Jiaotong University School of Medicine from 2014 to 2019, with written informed consent (Table 1). The fresh specimens were stored at −80°C for analysis.

**TABLE 1:** Clinical information of HCC patients.

Sex	
Male	26
Female	17
Age (years)	
≤51	13
>51	30
Hepatitics status	
Yes	38
No	5
Liver cirrhosis	
Yes	34
No	9
Child‐Pugh score	
A	36
B	7
AFP (ng/mL)	
≤20	12
>20	31
Tumour size	
≤5	21
>5	22
Tumour number	
Single	36
Multiple	7
Vascular invasion	
Yes	7
No	36
Lymph node metastasis	
Yes	8
No	35

HCC cell lines HCCLM3 (LM), MHCC97‐H (Hi), MHCC97 (Ori) and MHCC97‐L (Lo) were kindly offered by Dr. Dong Xie, Shanghai Institutes for Biological Sciences. HCC cells were cultured in RPMI‐1640 or DMEM supplemented with 10% fetal bovine serum, 10 mg/mL penicillin‐G and 10 mg/mL streptomycin. All cells were incubated at 37°C in a humidified atmosphere containing 5% CO_2_.

### 2.2 Reagents

Trizol reagent was purchased from Invitrogen. Luciferin was purchased from Xenogen Biotechnology. Antibodies used for western blotting were as follows: KLF9 (Abcam; ab227920), E‐cadherin (BD Transduction; 610,182), β‐actin (Cell Signaling Technology; 12,620), CK8/18 (Cell Signaling Technology; 4546), vimentin (Cell Signaling Technology; 3932), N‐cadherin (Cell Signaling Technology; 4061), and CK14 (Cell Signaling Technology; 46,251).

### 2.3 Plasmid construction and transfection

The full length of KLF9 complementary DNA was cloned into the pENTR‐1A vector at appropriate sites to generate the KLF9 overexpression vector. The truncated KLF9 (KLF9‐Δ) was constructed using the KLF9 cDNA without the nucleotides of 519–591. shRNA constructs targeting human KLF9 were shKLF9‐1 (AGTGATTCTGGGCCCTTTATG), shKLF9‐2 (CCCAGTGTCTGGTTTCCATTT). The pLKO‐puro/PLKO‐blast vector was used to express shRNAs. Lentivirus‐packaging plasmids and KLF9 shRNA or overexpression plasmids were transfected into 293T cells. The supernatant containing viral particles was collected and filtered with a 0.45‐μm filter (Millipore) and then used to infect HCC cells. Stable cells were selected by puromycin or blasticidin and used for future experiments.

### 2.4 Cell growth assay

HCC cells were plated in 100 μL of culture medium per well in 96‐well plates. Cell viability was measured with the CCK‐8 assay performed as previously described.^9^

### 2.5 Transwell assay

Transwell Assay was performed as previously described.^10^ The invasion assay was conducted using a Matrigel‐coated transwell chamber.

### 2.6 Xenograft study

Designated HCC cells (1 × 10^6^cells) were injected into dorsal flanks of 6–8‐week‐old nude mice. Tumours were measured over time with a calliper, and the tumour volume was calculated by the formula, volume = 0.5 × length × width^2^. All animals were randomized by weight. Mice were sacrificed and dissected at the endpoint. Xenografts were weighed after dissection. For experimental metastasis assays, 1 × 10^6^ designated HCC cells labelled with a luciferase reporter gene were injected into the left ventricle of the nude mice. Imaging metastasis in the mice was performed using an IVIS system over time, as previously described.^11^ For orthotopic metastasis assays, 1 × 10^6^ designated HCC cells labelled with a luciferase reporter gene were injected into the left lobe of the liver in the nude mice. The lungs of the animals were resected at a designated time, and the lung metastatic lesions were measured by an IVIS system and further analysed by H&E staining.

### 2.7 Chromatin immunoprecipitation (ChIP)

ChIP assay was conducted according to a previous report.^12^

### 2.8 Statistical analysis and reproducibility

All data are expressed as mean ± standard error (SE) if not indicated otherwise. All results, including western blotting, PCR and transwell assay, are representative of at least three independent experiments. Student's *t*‐test (two‐tailed) was used to compare two groups of data. A value of *P* < 0.05 was considered significant. A log‐rank test was applied for survival analysis.

## 3 RESULTS

### 3.1 KLF9 is downregulated in metastatic HCC cells and correlates with metastatic capabilities

**FIGURE 1:**
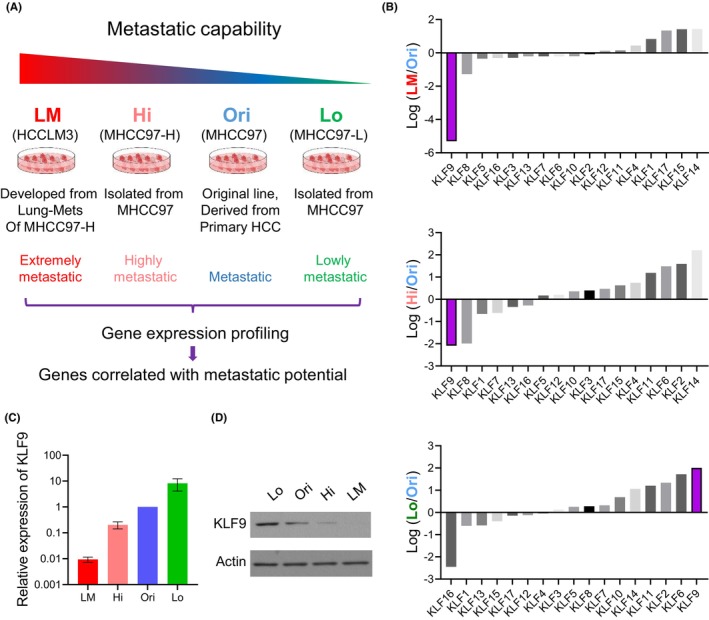
KLF9 is downregulated in metastatic liver cancer cells. (A) Relative metastatic capability of four established HCC cell lines. (B) Real‐time RT‐PCR showing the relative expression level of KLF family members in three HCC cell lines with differential metastatic capability normalized to MHCC97(Ori)cell line, which was moderately metastatic. (C) Real‐time RT‐PCR showing the relative expression level of KLF9 in three HCC cell lines with differential metastatic capability normalized to MHCC97(Ori)cell line. (D) Western blotting showing the expression level of KLF9 protein in four established HCC cell lines; Actin was used as loading control. Data are represented as mean ± SEM or the mean alone.

To identify the KLF family member that could regulate HCC metastasis, we first conducted gene expression profiling on the MHCC97 cell series. The MHCC97 series was established by a set of in vivo selections using the original, primary MHCC97 cells (Ori), aiming to establish a series of liver cancer cells with a continuous spectrum of metastatic capacities. With these efforts, three progeny clones were retrieved and propagated, namely MHCC97‐L (Lo), MHCC97‐H (Hi) and HCCLM3 (LM).^13^, ^14^ The metastatic capacity of these four lines is described in Figure 1A. By analysing the microarray analysis on these four lines, we found while most of the KLF family members were not significantly changed among these lines, the gene expression of KLF9 was negatively correlated with the metastatic potential of HCC cells (Figure 1B,C). As controls, the expression of other members of the KLF family, including KLF8, KLF14 and KLF6, was not altered in the MHCC97 cell series (Figure S1A–C). Consistent with the change in the mRNA level, the protein level of KLF9 was also increased while the metastatic ability of liver cancer cells weakened (Figure 1D). As controls, the expression of Slug, a pro‐metastasis gene, was increased in the highly metastatic lines, while Ecad‐, a classical antimetastasis protein was reduced in the highly metastatic lines (Figure S1D,E). The interesting anticorrelation between KLF9 expression and liver cancer metastasis suggested that KLF9 may be a negative regulator in liver cancer metastasis.

### 3.2 KLF9 suppresses migration and invasion of HCC cells in vitro

**FIGURE 2:**
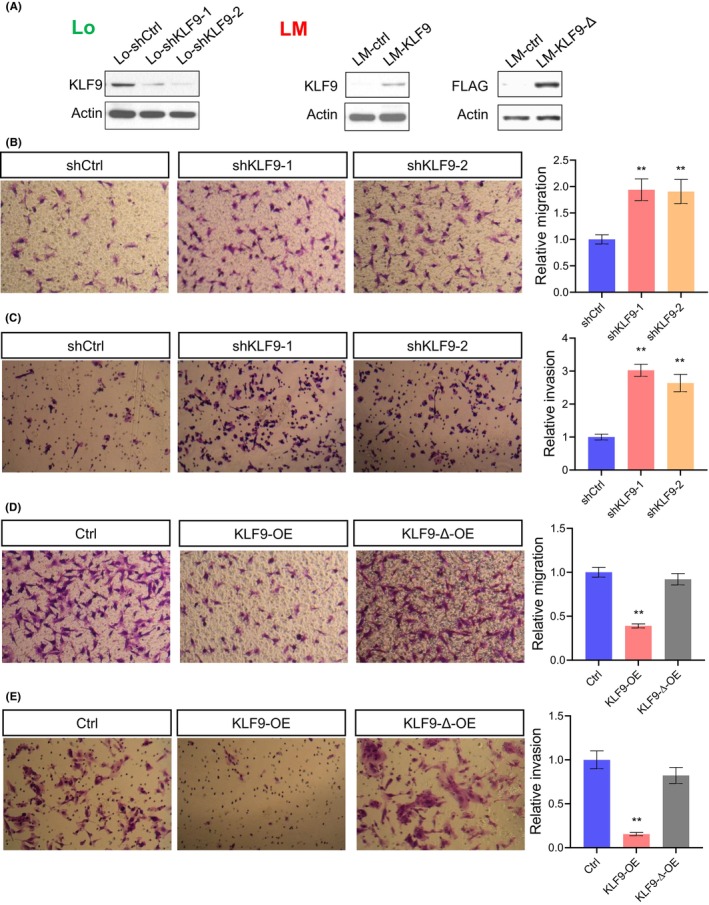
KLF9 suppresses migration and invasion of liver cancer. (A) Western blot verifying the knockdown and overexpression of full‐length KLF9 or truncated KLF9 (KLF9‐Δ) in the Lo (MHCC97‐L) cell line and LM (HCCLM3) cell line, respectively. (B) and (C) Transwell assay results showing the effects of KLF9 suppression on migration and invasion of the Lo cancer cells. The invasion assay was conducted using a Matrigel‐coated transwell chamber. (D) and (E) Transwell assay results showing the effects of overexpression of full‐length (KLF9‐OE) or truncated KLF9 (KLF9‐Δ‐OE) on migration and invasion of the LM cancer cells. * means *P* < 0.05, ** means *P* < 0.01. Data are represented as mean ± SEM or the mean alone. Illustration: Lo‐shCtrl, Lo‐shKLF9‐1 and Lo‐shKLF9‐2 refer to Lo cells transduced with a scramble shRNA or hairpins targeting KLF9; LM‐ctrl means LM cancer cell transfected with control vector, LM‐KLF9/KLF9‐OE means LM cell clones with stable forced expression of KLF9.

To study the role of KLF9 in liver cancer metastasis, we first tested the functions of KLF9 in cell migration and invasion in vitro. By use of two independent shRNAs, we inhibited the expression of KLF9 in MHCC97‐L (Lo) cells, which have relatively high expression of KLF9 (Figure 2A). In addition to the shRNA‐mediated loss‐of‐function study, we generated a KLF9‐overexpressing cell line in the HCCLM3 cells (LM), which have the lowest expression of KLF9 but highest metastatic potential (Figure 2A). To help validate the role of the transcriptional activity of KLF9, we also generated a truncated form of KLF9 (KLF9‐Δ), in which the C2H2 type 2 Zinc finger domain of the KLF9 protein was removed (Figure 2A, see methods for details).

By use of a transwell migration assay and a Matrigel‐coated invasion assay, we found that knockdown of KLF9 dramatically enhanced cell migration and invasion in the Lo cells (Figure 2B,C). A similar phenomenon can be observed in another cell line Ori (Figure S2A). On the contrary, forced expression of the full length KLF9 impaired cell motility and invasiveness in the LM cells (Figure 2D,E) and the MHCC97‐H (Hi) cells (Figure S2B). However, the KLF9 protein that loses its central DNA binding domain cannot affect cell migration or invasion, indicating that the role of KLF9 in cell motility and invasion depends on its transcription activity (Figure 2D,E). Nevertheless, we have to point out that KLF9 does not affect the cell growth of HCC cells (Figure S3). Thus, we conclude that KLF9 suppresses migration and invasion but not the proliferation of metastatic HCC cells.

### 3.3 KLF9 inhibits metastasis of HCC cells in vivo

**FIGURE 3:**
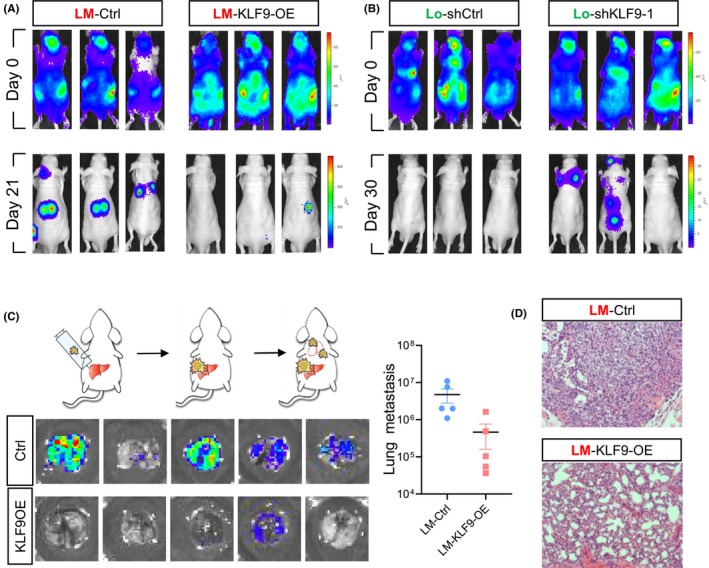
KLF9 inhibits metastasis of liver cancer in vivo. (A) Xenograft studies and bioluminescence imaging showing that injection of LM (HCCLM3) cells with KLF9 overexpression leads to fewer and smaller metastatic tumour lesions in nude mice compared to control LM cells. (B) Xenograft studies and bioluminescence imaging showing that injection of Lo (MHCC97‐L) cells with KLF9 knockdown leads to more and greater metastatic tumour lesions in nude mice compared to control Lo cells. (C) The LM Liver cancer cells with or without KLF9 overexpression were injected into the liver of nude mice to allow lung metastasis formation. The lung of the implanted animals was harvested, and bioluminescence measurement was conducted. (D) H&E staining on the lung tissue of C was conducted to verify lung metastasis. Data are represented as mean ± SEM or the mean alone.

We next investigated if KLF9 affects tumour growth and metastasis of liver cancer in vivo. Consistent with its role in cell growth in vitro, altered expression of KLF9 only mildly affects the tumour growth of metastatic liver cancer cells in vivo (Figure S4). To examine the role of KLF9 in metastasis of liver cancer cells, we first applied an intracardiac injection‐based experimental metastasis assay in both LM and Lo cells. As we expected, forced expression of KLF9 in the highly metastatic cell line LM effectively suppressed metastasis in vivo (Figure 3A), while knockdown of KLF9 dramatically enhances the metastatic potential of Lo cells (Figure 3B). To further validate the function of KLF9 in HCC metastasis, we applied an orthotopic metastasis assay, which can more faithfully indicate and represent the metastatic capacity of liver cancers. We injected both the LM‐KLF9‐OE and control LM cells into the liver of nude mice. As reported previously,^14^ implantation of LM cells in the liver generated metastasis in lung within 4 weeks. We found while the control LM cells can effectively form lung metastasis, overexpression of KLF9 dramatically abolished lung metastasis (Figure 3C,D). Collectively, these data demonstrated that KLF9 inhibits metastasis of HCC in vivo.

### 3.4 KLF9 reverses the EMT program in HCC cells

**FIGURE 4:**
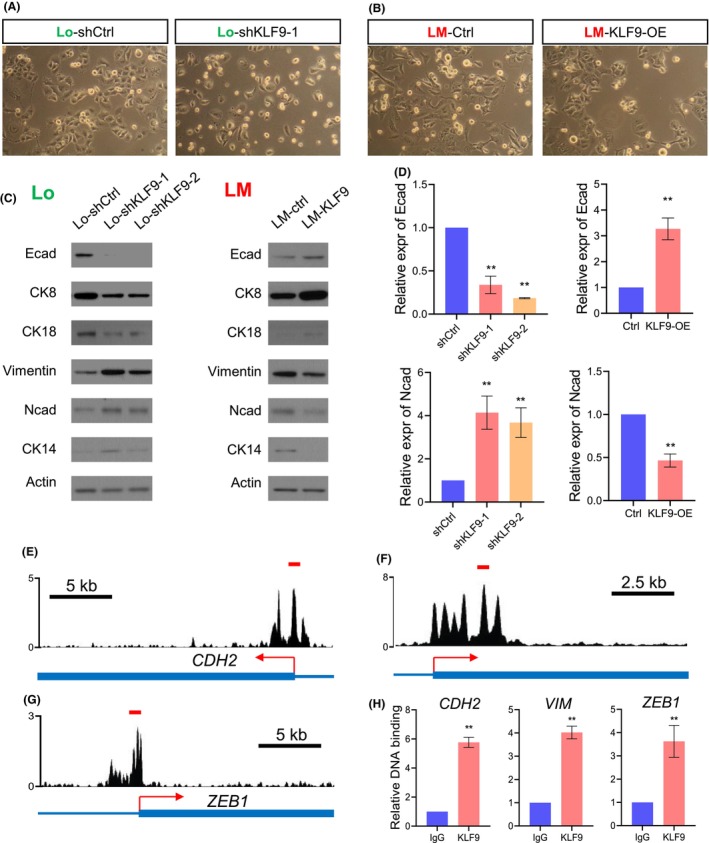
KLF9 promotes a MET program in liver cancer cells. (A) Cell morphology of Lo cells with or without KLF9 knockdown was captured and displayed. (B) Cell morphology of LM cells with or without KLF9 overexpression was captured and displayed. (C) Western blot showing knockdown of KLF9 in Lo cell line leads to a decrease of epithelial markers, including Ecad, CK‐8 and CK‐18, and an increase in mesenchymal markers including vimentin, Ncad and CK14 (left lane). Overexpression of KLF9 in the LM cells caused an increase in epithelial marker expression and loss of mesenchymal marker expression (right lane). (D) Real‐time RT‐PCR showing the effects of KLF9 knockdown or overexpression on mRNA level of Ecad and Ncad. (E) to (G) The KLF9‐bound peaks on the CDH2, VIM and ZEB1 genes of the GBM1B cells were adapted from Cistrome.org; the transcriptional start site of each gene was indicated in red. (H) ChIP was conducted in the HCCLM3 cells that overexpressed KLF9 using an anti‐KLF9 antibody. PCR assays were conducted to measure the enrichment of KLF9‐bound DNA in the indicated genes. The red lines in (E)–(G) indicate the locations of the amplified regions. * means *P* < 0.05, ** means *P* < 0.01. Data are represented as mean ± SEM or the mean alone.

We next wanted to interrogate the mechanism of how KLF9 regulates cell migration and metastasis. EMT is a pivot process that facilitates migration, invasion and metastasis of tumour cells.^4^, ^15^ Based on our findings that KLF9 could suppress migration and invasion, we hypothesized KLF9 might modulate EMT in HCC cells. Indeed, we found that knockdown of KLF9 in the Lo cells induces a mesenchymal morphologic change—occurrence of a spindle‐like shape and loose intercellular junction (Figure 4A). In contrast, overexpression of KLF9 in LM cells can induce an inverse alteration, such that the LM cells become rounder, flatter, of a tighter intercellular connection (Figure 4B). These changes suggest that expression of KLF9 can suppress EMT. Next, we examined the expression of a set of epithelial and mesenchymal markers. Consistent with the morphologic change, knockdown of KLF9 leads to decreased expression of epithelial marker protein, including E‐cadherin, CK‐8 and CK‐18, as well as an increase in mesenchymal marker protein including vimentin, N‐cadherin and CK14 (Figure 4C, left lane). In stark contrast, overexpression of KLF9 leads to complete opposite alterations (Figure 4C, right lane). Similar changes can be confirmed by testing at the RNA level (Figure 4D). These results suggested that KLF9 suppresses the EMT program in HCC cells.

As a TF, KLF9 was known to repress transcription by direct binding to the promoter regions of target genes.^16^, ^17^, ^18^ Therefore, we wondered if KLF9 repressed mesenchymal gene expression by direct binding to their genomic regions. By interrogating the Cistrome ChIP‐seq database, we identified that at least two previous studies investigated the genomic binding of KLF9 in two independent cell lines (GBM1B and 293T; CistromeDB 49,951, 62,637). We found that the promoter region of *CDH2* had a significant signal of KLF9 binding in both GBM1B and 293T cells (Figure 4E; Figure S5A). In addition to *CDH2*, two additional mesenchymal genes, *VIM* and *ZEB1*, also exhibited very strong KLF9 binding at their promoter regions (Figure 4F,G; Figure S5B,C). While this was consistent in two lines, we next wanted to confirm these findings in the HCC cells we used. Consistent with our hypothesis, we found that KLF9 bind to the mesenchymal genes in the LM cells that overexpressed KLF9 (Figure 4H, ChIP‐PCR), which may be the mechanism of how KLF9 represses their expression thus suppressing metastasis. Together, we found that KLF9 can suppress the EMT programme to modulate HCC metastasis.

### 3.5 Slug negatively regulates the expression of KLF9

**FIGURE 5:**
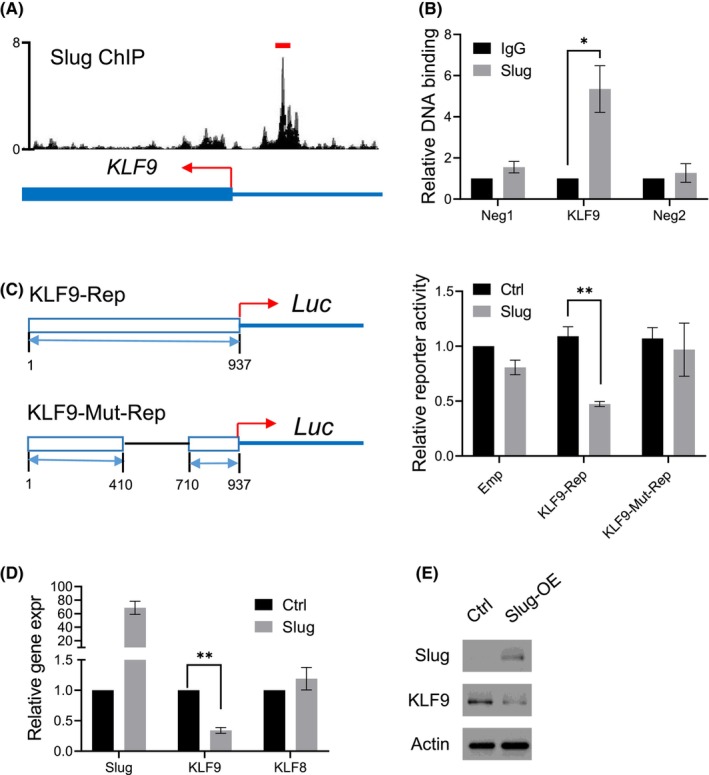
Slug negatively regulates KLF9 transcription. (A) The Slug‐bound peak on the KLF9 gene was adapted from Cistrome.org; the transcriptional start site of KLF9 was indicated in red. The region that was cloned and used in the following reporter assays was indicated by a red line. (B) ChIP was conducted in the HCCLM3 cells using an anti‐Slug antibody. PCR assays were conducted to measure the enrichment of Slug‐bound DNA in three indicated regions, including the Slug‐peak region and two flanking control regions. (C) A control pGL3‐promoter reporter (Emp), a pGL3‐promoter reporter inserted with the Slug‐bound KLF9 upstream (KLF9‐Rep), and a truncated form the KLF‐Rep (KLF9‐Mut‐Rep) were transfected into control 293T and 293T cells overexpressing Slug, together with a Renilla control reporter. Dual Luciferase assays were conducted to measure the luciferase activity of each condition. Left, schematic of the design of the KLF9 reporters. (D) qPCR showing the mRNA level of Slug, KLF9 and KLF8 in control 293T cells and 293T cells overexpressing Slug. (E) Western blotting showing the protein level of Slug and KLF9 in control 293T cells and 293T cells overexpressing Slug.

To further investigate the mechanism that regulates the expression of KLF9 in cancer, we again turned to the ChIP‐seq database to look for possible transcription factors that can bind to the regulatory regions of the KLF9. In particular, we were interested in the classical EMT TFs, including Twist, Snail and Slug. While there were no obvious binding peaks of Twist or Snail at the upstream regions of the KLF9 gene, we found that there was a very strong Slug‐bound peak at the 5 kb upstream of the KLF9 transcription start site (Figure 5A). Since the Slug ChIPseq was conducted in 293T cells, we wanted to confirm if this Slug‐binding was conserved in HCC cells. By use of a Slug antibody, we pulled down the Slug‐bound chromatin elements from the LM cells and interrogated the KLF9 gene region by PCR assays. We found that the Slug antibody can significantly pull down the designated 5′ upstream elements of the KLF9 gene but not the flanking control elements (Figure 5B). These results suggested that the Slug protein can directly bind to a specific region of the 5′ upstream regulatory element of the KLF9 gene.

Next, we determined if the binding of Slug to the KLF9 promotor region can affect the latter's transcription. We cloned the Slug‐bound sequence into a pGL3‐promoter luciferase reporter (KLF9‐Rep, Figure 5C left), which contains a basal transcription of the luciferase protein. Without insertion of the Slug‐bound element, overexpression of the Slug protein did not alter the transcriptional activity of the reporter. In contrast, overexpression of Slug can dramatically suppress the transcriptional activity of the KLF9 reporter, indicating that the binding of Slug to its regulatory element on the KLF9 gene can directly inhibit KLF9 transcription (Figure 5C, right). To confirm the binding of Slug to the regulatory region of KLF9 was essential, we made a truncated form of the KLF9 reporter (KLF9‐Mut‐Rep, Figure 5C left). In contrast to the KLF9‐Rep, the reporter that lacks the Slug‐binding element did not respond to Slug‐overexpression (Figure 5C right). To verify the regulation of Slug on KLF9, we measured the expression level of KLF9 in 293T cells overexpressing Slug. We found that the RNA and protein product of the KLF9 gene was significantly decreased upon Slug overexpression (Figure 5D,E). As a control, the expression of KLF8, another member of the KLF family, was not altered upon Slug expression. Together, these results suggested that Slug can directly suppress the expression of KLF9.

### 3.6 KLF9 is downregulated in metastatic HCC and correlates with survival

**FIGURE 6:**
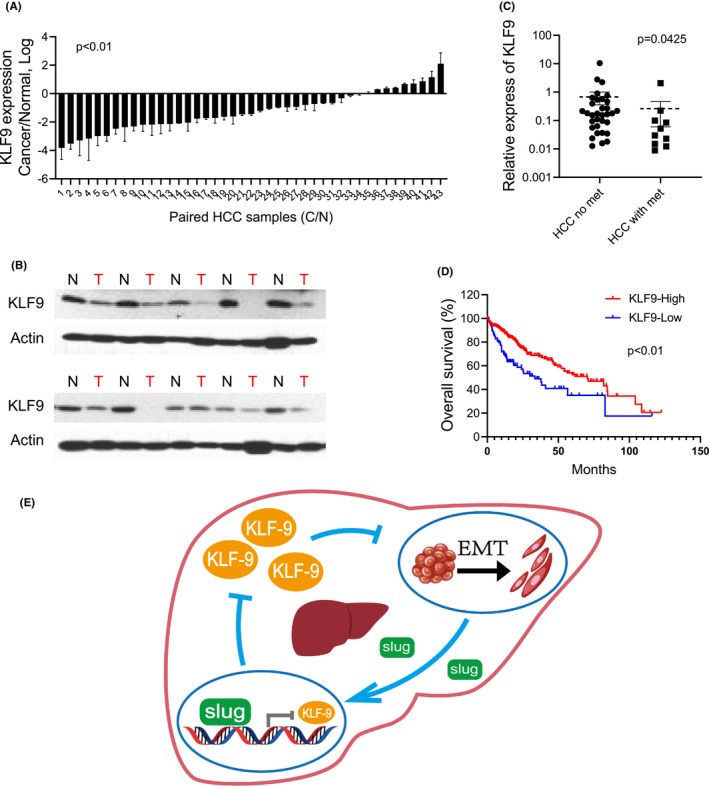
KLF9 is downregulated in metastatic HCC and correlates with survival. (A) Real‐time RT‐PCR results showing the relative expression of KLF9 mRNA in 43 HCC tumour samples and their matched normal tissues. (B) Western blotting showing the expression of KLF9 protein in 10 HCC samples and paired normal tissues; Actin was used as loading control. T, tumour samples; N, paired normal tissues. (C) Real‐time RT‐PCR results showing the relative expression of KLF9 mRNA differs in HCC patients with or without metastasis. (D) Kaplan–Meier survival analysis showing the overall survival probability of HCC patients with high/low KLF9 mRNA expression by month (data were adapted from the TCGA database). (E) Schematic representation of the negative feedback loop between KLF9 and EMT program in liver cancer that promotes metastasis. Data are represented as mean ± SEM or the mean alone.

Finally, we validated the clinical relevance of KLF9 in live cancer using clinical tissue samples. We measured the transcriptional level of KLF9 by real‐time RT‐PCR in 43 pairs of human HCC tumour samples and matched normal tissues (Table 1). We observed a decrease in KLF9 mRNA level in 34 of 43 (79.1%) HCC cancer samples compared to their paired normal tissues (Figure 6A). Similarly, a decrease in KLF9 protein level was also found in HCC cancer tissues compared to paired normal tissues as shown by western blot (Figure 6B). More strikingly, the expression of KLF9 was further downregulated in the patients who developed intrahepatic metastasis at diagnosis compared to patients who did not have metastatic diseases (Figure 6C), further suggesting an antimetastasis role of KLF9 in liver cancer. Lastly, we found that the expression level of KLF9 was positively correlated with the overall survival of HCC patients (TCGA, Figure 6D). Taking together, we demonstrated that KLF9 is downregulated in metastatic HCC and correlates with patients' overall survival.

## 4 DISCUSSION

HCC is a malignant epithelial tumour with high incidence and mortality. Unfortunately, a considerable proportion of HCC patients have developed metastasis at diagnosis. Although, in recent years, multitarget tyrosine kinase inhibitors (such as sorafenib and lenvatinib), bevacizumab and immune checkpoint inhibitors have been employed in systemic therapy of HCC, the median survival time of advanced HCC did not exceed 2 years in clinical trials up to date with the efforts made.^19^, ^20^, ^21^, ^22^ Therefore, exploring novel regulating factors of HCC metastasis will shed light on developing treatment targets and improving long‐term outcomes for patients with metastatic HCC.

Tumour metastasis is a multistep process, regulated or driven by different mechanisms, including EMT, metastasis‐associated gene abnormalities and tumour microenvironment alterations.^4^, ^23^, ^24^ In addition, epigenetic modification, including long noncoding RNAs (lncRNAs) and chromatin remodelling factors, also contribute to HCC metastasis.^25^, ^26^, ^27^ EMT plays a critical role early in HCC metastasis when cells lose intercellular contacts and acquire increased motility to spread. EMT‐TFs such as Snail, Twist and ZEB are responsible for executing EMT, and known factors impinging on EMT promotion include TGF‐β, c‐Met and various micro‐RNAs.^28^ Clinical trials have been carried out to test medications targeting EMT regulators in HCC, such as TGFR1 inhibitors and c‐MET inhibitors, but only a small portion of patients with specific genetic profiles benefit from these therapies.^29^, ^30^, ^31^ Hence, further studies are required to identify novel regulators of EMT and metastasis in HCC as potential therapeutic targets. Our findings revealed a new mechanism we may exploit to target the EMT program and metastasis (Figure 6E).

As one of the largest TF families, the KLF family has been reported to be engaged in cancer progression in cancers of various origins. Therefore, it is crucial to dissect the tissue‐specific function of the KLFs in cancer. As the most well‐known factor of the KLF family, the function of KLF4 in cancer has been extensively studied. For instance, KLF4 functions as an oncogene in B non‐Hodgkin lymphoma but appears as a tumour suppressor in several epithelial cancers, such as lung cancer and cancers in the digestive tract.^6^ Mechanistic studies revealed that KLF4 might regulate EMT by repressing the promoter of Slug—an EMT transcription factor.^32^ In addition, KLF5 could regulate stemness, cell proliferation, apoptosis, autophagy or migration of cancer cells, depending on the types of cancer.^33^ In HCC, KLF5 negatively correlates with patients' prognosis and may promote HCC growth and metastasis by activating PI3K/AKT/Snail signalling.^34^ Interestingly, the role of KLF5 on EMT may depend on the p53 status, as KLF5 inhibits ZEB2 expression and EMT by inducing miR‐192 when p53 is inactivated, but not in HCC cell line with wild‐type p53.^35^ Our findings further enrich the complicated connection between the KLFs and cancer by establishing a tumour‐suppressive role of KLF9 in HCC progression.

Progression into a mesenchymal cell state is regarded as a phenotypic marker of cancer stem cells in a variety of cancers, including breast cancer and ovarian cancer. Several previous studies have revealed that KLF9 could regulate tumour initiation in other cancer types, in concert with our perspective that KLF9 works as an EMT regulator. For instance, reduction of KLF9 could facilitate stemness in ovarian cancer via Notch1/slug signalling.^36^ In addition, KLF9 functions as a haploinsufficient suppressor of colon tumorigenesis in Apc^Min/+^ mice by inhibition of interferon‐related signalling.^37^ Zhou QQ et al. reported that SNX5 could inhibit TGF‐β‐induced migration, invasion and EMT in clear cell renal cell carcinoma cells, and KLF9 directly binds to the SNX5 promoter and upregulates SNX5 transcription, thus inhibiting migration and EMT.^38^ However, in oral squamous cell carcinoma (OSCC), Wang CY et al. revealed a LINC00664/miR‐411‐5p/KLF9‐positive feedback loop that could promote migration, invasion and EMT of OSCC cells.^39^ These controversial findings mentioned above suggest that the tissue‐dependent role of KLF9 needs to be further investigated. Effects of KLF9 on liver cancer metastasis and EMT are still elusive, and we originally uncovered that KLF9 functionally suppresses HCC metastasis and EMT in this study, which will be a novel therapeutic target for drug development.

Among all the important features of cancer cells, we specifically identified that KLF9 suppresses HCC metastasis, the deadliest stage of this disease. In addition, we found that KLF9 negatively regulates invasion and metastasis of HCC but not affecting cell growth or tumour growth. These findings precisely dissociate the anti‐growth and anti‐invasion/metastasis roles of KLF9. A previous report has shown that KLF9 inhibits cell proliferation in HCC cells, seemingly controversial in our studies. However, this report identified that the cell growth inhibitory effect of KLF9 depends on the expression of a wild‐type p53 protein.^40^ Interestingly, the MHCC97 cells we used in this study were p53‐null, which abolishes the p53‐dependent function of KLF9. In fact, this exciting relationship between KLF9's function and the status of essential oncogenes or tumour suppressors is reminiscent of the findings of KLF5, suggesting that this could be, to some level, a common phenomenon of the KLF family. It would be interesting to dig into the mechanisms of other KLFs from this perspective. Further comparison of KLF9's function between different cancer types could help unveil the fundamental mechanisms of KLF9 underlying its functions in cancer cells.

## AUTHOR CONTRIBUTIONS

**Tao Wang:** Data curation (equal); formal analysis (equal); investigation (equal). **Limin Feng:** Data curation (equal); investigation (equal); validation (equal). **Zhong Shi:** Data curation (equal); investigation (equal); methodology (equal). **Lixian Yang:** Data curation (equal); formal analysis (equal); investigation (equal). **Xiaofu Yu:** Data curation (equal); investigation (equal); methodology (equal). **Jirui Sun:** Data curation (equal); investigation (equal); methodology (equal). **Jinku Zhang:** Data curation (equal); investigation (equal); resources (equal). **Weilin Wang:** Data curation (equal); funding acquisition (equal); investigation (equal). **Jinsong Wu:** Conceptualization (equal); formal analysis (equal); funding acquisition (equal). **Yuxiong Feng:** Conceptualization (equal); funding acquisition (equal); investigation (equal); supervision (equal).

## FUNDING INFORMATION

This study was supported by grants from the Ministry of Science and Technology of the People's Republic of China (2020YFA0803300), the National Natural Science Foundation of China (82072650, 31871369 and 32270783), the Key Research and Development Program of Zhejiang Province (2021C03121), Zhejiang Provincial Natural Science Foundation of China (LY22H160014), Zhejiang University Basic Research Fund (226‐2022‐00037) and Baoding Research Project Fund (2041ZF084).

## CONFLICT OF INTEREST STATEMENT

The authors have no conflict of interest.

## Supporting information


Figure S1–S5

## Data Availability

All raw data of this manuscript is available upon request to the corresponding authors.

## References

[1] Sung H , Ferlay J , Siegel RL , et al. Global cancer statistics 2020: GLOBOCAN estimates of incidence and mortality worldwide for 36 cancers in 185 countries. CA Cancer J Clin. 2021;71:209‐249. 33538338 10.3322/caac.21660

[2] Balogh J , Victor D III , Asham EH , et al. Hepatocellular carcinoma: a review. J Hepatocell Carcinoma. 2016;3:41‐53. 27785449 10.2147/JHC.S61146 PMC5063561

[3] Llovet JM , Montal R , Sia D , Finn RS . Molecular therapies and precision medicine for hepatocellular carcinoma. Nat Rev Clin Oncol. 2018;15:599‐616. 30061739 10.1038/s41571-018-0073-4 PMC12452113

[4] Mittal V . Epithelial mesenchymal transition in tumor metastasis. Annu Rev Pathol. 2018;13:395‐412. 29414248 10.1146/annurev-pathol-020117-043854

[5] McConnell BB , Yang VW . Mammalian Kruppel‐like factors in health and diseases. Physiol Rev. 2010;90:1337‐1381. 20959618 10.1152/physrev.00058.2009 PMC2975554

[6] Lu XJ , Shi Y , Chen JL , Ma S . Kruppel‐like factors in hepatocellular carcinoma. Tumour Biol. 2015;36:533‐541. 25652467 10.1007/s13277-015-3127-6

[7] Takahashi K , Tanabe K , Ohnuki M , et al. Induction of pluripotent stem cells from adult human fibroblasts by defined factors. Cell. 2007;131:861‐872. 18035408 10.1016/j.cell.2007.11.019

[8] Park IH , Zhao R , West JA , et al. Reprogramming of human somatic cells to pluripotency with defined factors. Nature. 2008;451:141‐146. 18157115 10.1038/nature06534

[9] Zhao JS , Shi S , Qu HY , et al. Glutamine synthetase licenses APC/C‐mediated mitotic progression to drive cell growth. Nat Metab. 2022;4:239‐253. 35145325 10.1038/s42255-021-00524-2

[10] Feng YX , Jin DX , Sokol ES , Reinhardt F , Miller DH , Gupta PB . Cancer‐specific PERK signaling drives invasion and metastasis through CREB3L1. Nat Commun. 2017;8:1079. 29057869 10.1038/s41467-017-01052-y PMC5651903

[11] Gao H , Yin FF , Guan DX , et al. Liver cancer: WISP3 suppresses hepatocellular carcinoma progression by negative regulation of beta‐catenin/TCF/LEF signalling. Cell Prolif. 2019;52:e12583. 30793395 10.1111/cpr.12583 PMC6536422

[12] Li W , Notani D , Ma Q , et al. Functional roles of enhancer RNAs for oestrogen‐dependent transcriptional activation. Nature. 2013;498:516‐520. 23728302 10.1038/nature12210 PMC3718886

[13] Tian J , Tang ZY , Ye SL , et al. New human hepatocellular carcinoma (HCC) cell line with highly metastatic potential (MHCC97) and its expressions of the factors associated with metastasis. Br J Cancer. 1999;81:814‐821. 10555751 10.1038/sj.bjc.6690769 PMC2374300

[14] Li Y , Tang Y , Ye L , et al. Establishment of a hepatocellular carcinoma cell line with unique metastatic characteristics through in vivo selection and screening for metastasis‐related genes through cDNA microarray. J Cancer Res Clin Oncol. 2003;129:43‐51. 12618900 10.1007/s00432-002-0396-4 PMC12161897

[15] Dong Y , Zheng Q , Wang Z , et al. Higher matrix stiffness as an independent initiator triggers epithelial‐mesenchymal transition and facilitates HCC metastasis. J Hematol Oncol. 2019;12:112. 31703598 10.1186/s13045-019-0795-5 PMC6839087

[16] Kang L , Lai MD . BTEB/KLF9 and its transcriptional regulation. Yi Chuan. 2007;29:515‐522. 17548317 10.1360/yc-007-0515

[17] Bai XY , Li S , Wang M , et al. Kruppel‐like factor 9 down‐regulates matrix metalloproteinase 9 transcription and suppresses human breast cancer invasion. Cancer Lett. 2018;412:224‐235. 29107105 10.1016/j.canlet.2017.10.027

[18] Li Y , Sun Q , Jiang M , et al. KLF9 suppresses gastric cancer cell invasion and metastasis through transcriptional inhibition of MMP28. FASEB J. 2019;33:7915‐7928. 30913394 10.1096/fj.201802531R

[19] Yau T , Kang YK , Kim TY , et al. Efficacy and safety of nivolumab plus ipilimumab in patients with advanced hepatocellular carcinoma previously treated with sorafenib: the CheckMate 040 randomized clinical trial, JAMA. Oncologia. 2020;6:e204564. 10.1001/jamaoncol.2020.4564 PMC7530824 33001135

[20] Cheng AL , Qin S , Ikeda M , et al. Updated efficacy and safety data from IMbrave150: Atezolizumab plus bevacizumab vs. sorafenib for unresectable hepatocellular carcinoma. J Hepatol. 2022;76:862‐873. 34902530 10.1016/j.jhep.2021.11.030

[21] Dual immunotherapy makes strides against HCC. Cancer Discov. 2022;12:OF1. 10.1158/2159-8290.CD-NB2022-0008 35105553

[22] Ren Z , Xu J , Bai Y , et al. Sintilimab plus a bevacizumab biosimilar (IBI305) versus sorafenib in unresectable hepatocellular carcinoma (ORIENT‐32): a randomised, open‐label, phase 2‐3 study. Lancet Oncol. 2021;22:977‐990. 34143971 10.1016/S1470-2045(21)00252-7

[23] Yang LY , Luo Q , Lu L , et al. Increased neutrophil extracellular traps promote metastasis potential of hepatocellular carcinoma via provoking tumorous inflammatory response. J Hematol Oncol. 2020;13:3. 31907001 10.1186/s13045-019-0836-0 PMC6945602

[24] Lu S , Yang LX , Cao ZJ , Zhao JS , You J , Feng YX . Transcriptional control of metastasis by integrated stress response signaling. Front Oncol. 2021;11:770843. 34746012 10.3389/fonc.2021.770843 PMC8570279

[25] Li H , Chen Z , Zhang Y , et al. MiR‐4310 regulates hepatocellular carcinoma growth and metastasis through lipid synthesis. Cancer Lett. 2021;519:161‐171. 34303763 10.1016/j.canlet.2021.07.029

[26] Jiang H , Cao HJ , Ma N , et al. Chromatin remodeling factor ARID2 suppresses hepatocellular carcinoma metastasis via DNMT1‐snail axis. Proc Natl Acad Sci U S A. 2020;117:4770‐4780. 32071245 10.1073/pnas.1914937117 PMC7060681

[27] Zhu X , Pan H , Liu L . Long noncoding RNA network: novel insight into hepatocellular carcinoma metastasis (review). Int J Mol Med. 2021;48:134. 34013360 10.3892/ijmm.2021.4967 PMC8148093

[28] Giannelli G , Koudelkova P , Dituri F , Mikulits W . Role of epithelial to mesenchymal transition in hepatocellular carcinoma. J Hepatol. 2016;65:798‐808. 27212245 10.1016/j.jhep.2016.05.007

[29] Harding JJ , Do RK , Yaqubie A , et al. Phase 1b study of galunisertib and ramucirumab in patients with advanced hepatocellular carcinoma. Cancer Med. 2021;10:3059‐3067. 33811482 10.1002/cam4.3880 PMC8085979

[30] Jindal A , Thadi A , Shailubhai K . Hepatocellular carcinoma: etiology and current and future drugs. J Clin Exp Hepatol. 2019;9:221‐232. 31024205 10.1016/j.jceh.2019.01.004 PMC6477125

[31] Bouattour M , Raymond E , Qin S , et al. Recent developments of c‐met as a therapeutic target in hepatocellular carcinoma. Hepatology. 2018;67:1132‐1149. 28862760 10.1002/hep.29496 PMC5873445

[32] Lin ZS , Chu HC , Yen YC , Lewis BC , Chen YW . Kruppel‐like factor 4, a tumor suppressor in hepatocellular carcinoma cells reverts epithelial mesenchymal transition by suppressing slug expression. PLoS One. 2012;7:e43593. 22937066 10.1371/journal.pone.0043593 PMC3427336

[33] Luo Y , Chen C . The roles and regulation of the KLF5 transcription factor in cancers. Cancer Sci. 2021;112:2097‐2117. 33811715 10.1111/cas.14910 PMC8177779

[34] An T , Dong T , Zhou H , et al. The transcription factor Kruppel‐like factor 5 promotes cell growth and metastasis via activating PI3K/AKT/snail signaling in hepatocellular carcinoma. Biochem Biophys Res Commun. 2019;508:159‐168. 30473218 10.1016/j.bbrc.2018.11.084

[35] Sun L , Zhou X , Li Y , et al. KLF5 regulates epithelial‐mesenchymal transition of liver cancer cells in the context of p53 loss through miR‐192 targeting of ZEB2. Cell Adh Migr. 2020;14:182‐194. 32965165 10.1080/19336918.2020.1826216 PMC7553557

[36] Wang K , Liu S , Dou Z , Zhang S , Yang X . Loss of Kruppel‐like factor 9 facilitates stemness in ovarian cancer ascites‐derived multicellular spheroids via Notch1/slug signaling. Cancer Sci. 2021;112:4220‐4233. 34363722 10.1111/cas.15100 PMC8486214

[37] Brown AR , Simmen RC , Raj VR , Van TT , MacLeod SL , Simmen FA . Kruppel‐like factor 9 (KLF9) prevents colorectal cancer through inhibition of interferon‐related signaling. Carcinogenesis. 2015;36:946‐955. 26210742 10.1093/carcin/bgv104 PMC4573661

[38] Zhou Q , Li J , Ge C , Chen J , Tian W , Tian H . SNX5 suppresses clear cell renal cell carcinoma progression by inducing CD44 internalization and epithelial‐to‐mesenchymal transition. Mol Ther Oncolytics. 2022;24:87‐100. 35024436 10.1016/j.omto.2021.12.002 PMC8717386

[39] Wang C , Wang Q , Weng Z . LINC00664/miR‐411‐5p/KLF9 feedback loop contributes to the human oral squamous cell carcinoma progression. Oral Dis. 2021;29:672‐685. 34582069 10.1111/odi.14033

[40] Sun J , Wang B , Liu Y , et al. Transcription factor KLF9 suppresses the growth of hepatocellular carcinoma cells in vivo and positively regulates p53 expression. Cancer Lett. 2014;355:25‐33. 25242357 10.1016/j.canlet.2014.09.022

