# Supplementary material for: A negative feedback loop between KLF9 and the EMT program dictates metastasis of hepatocellular carcinoma
Source: J Cell Mol Med. 2023 Jul 3;27(16):2372–84. doi: 10.1111/jcmm.17823 (PMC10424290; doi:10.1111/jcmm.17823)

Fig.S1 Expression of KLF8, KLF14 and KLF6 in MHCC97 cell series

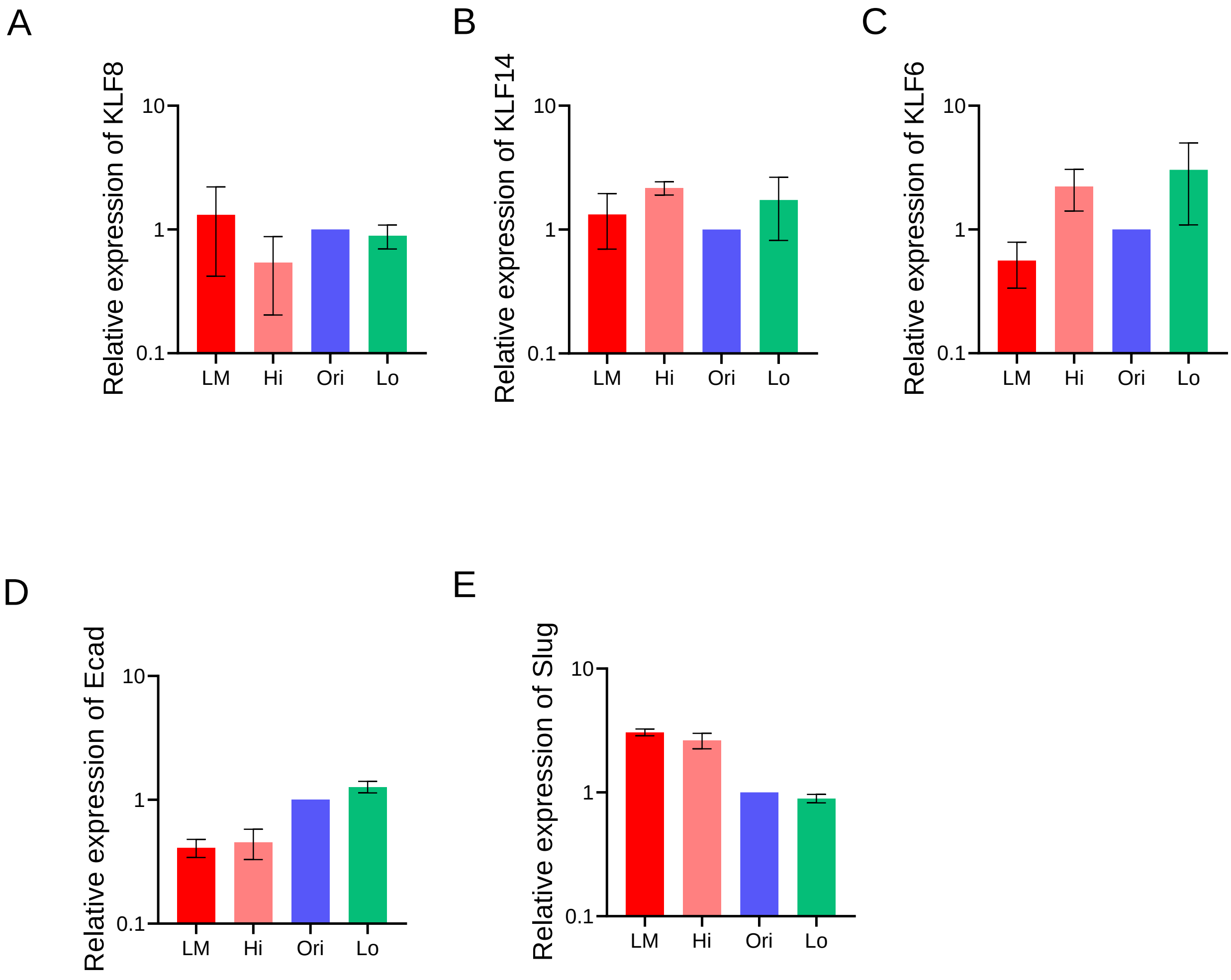

Fig.S2 KLF9 regulates migration in multiple HCC cells

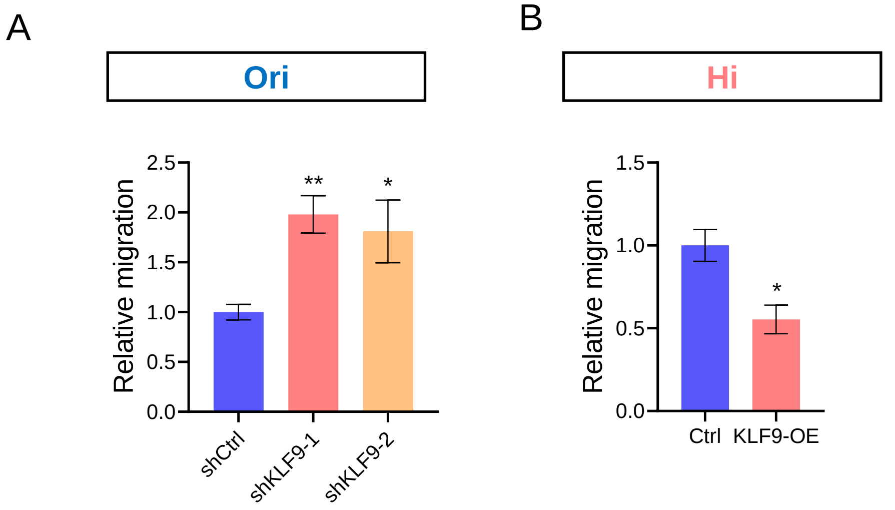

Fig.S3 KLF9 only mildly affects cell growth of HCC cells

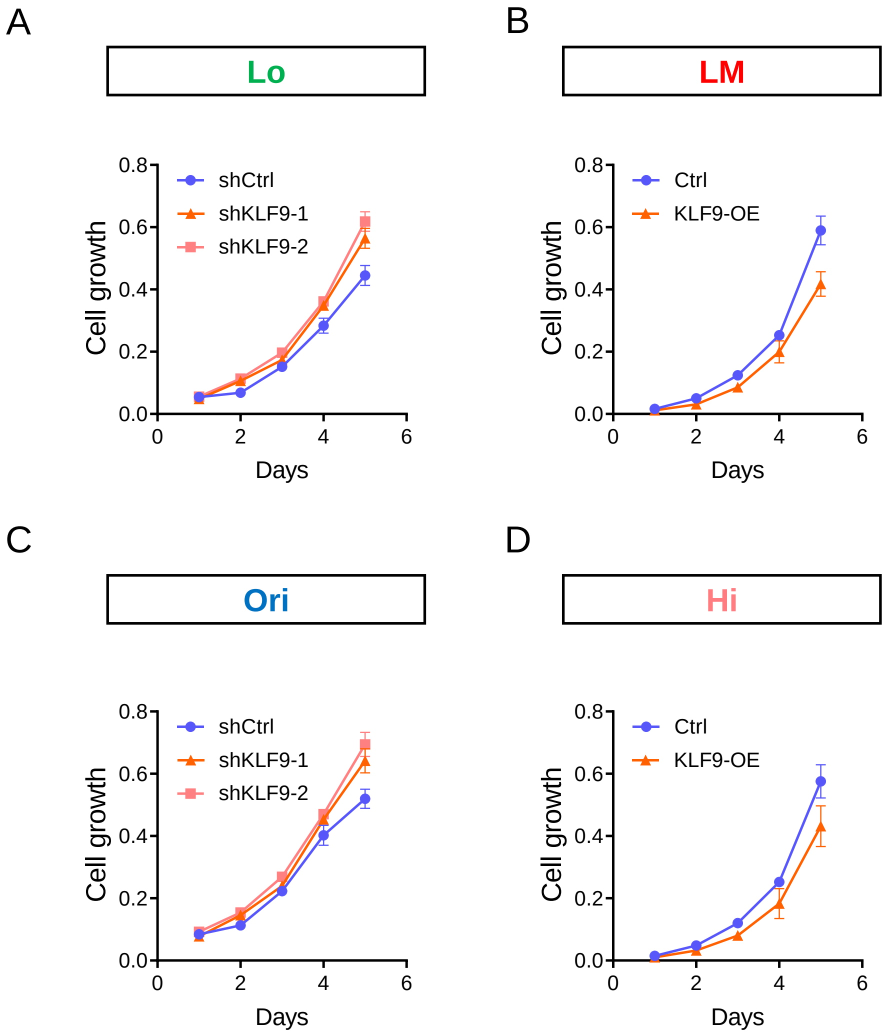

Fig.S4 KLF9 moderately regulates tumor growth of HCC cells

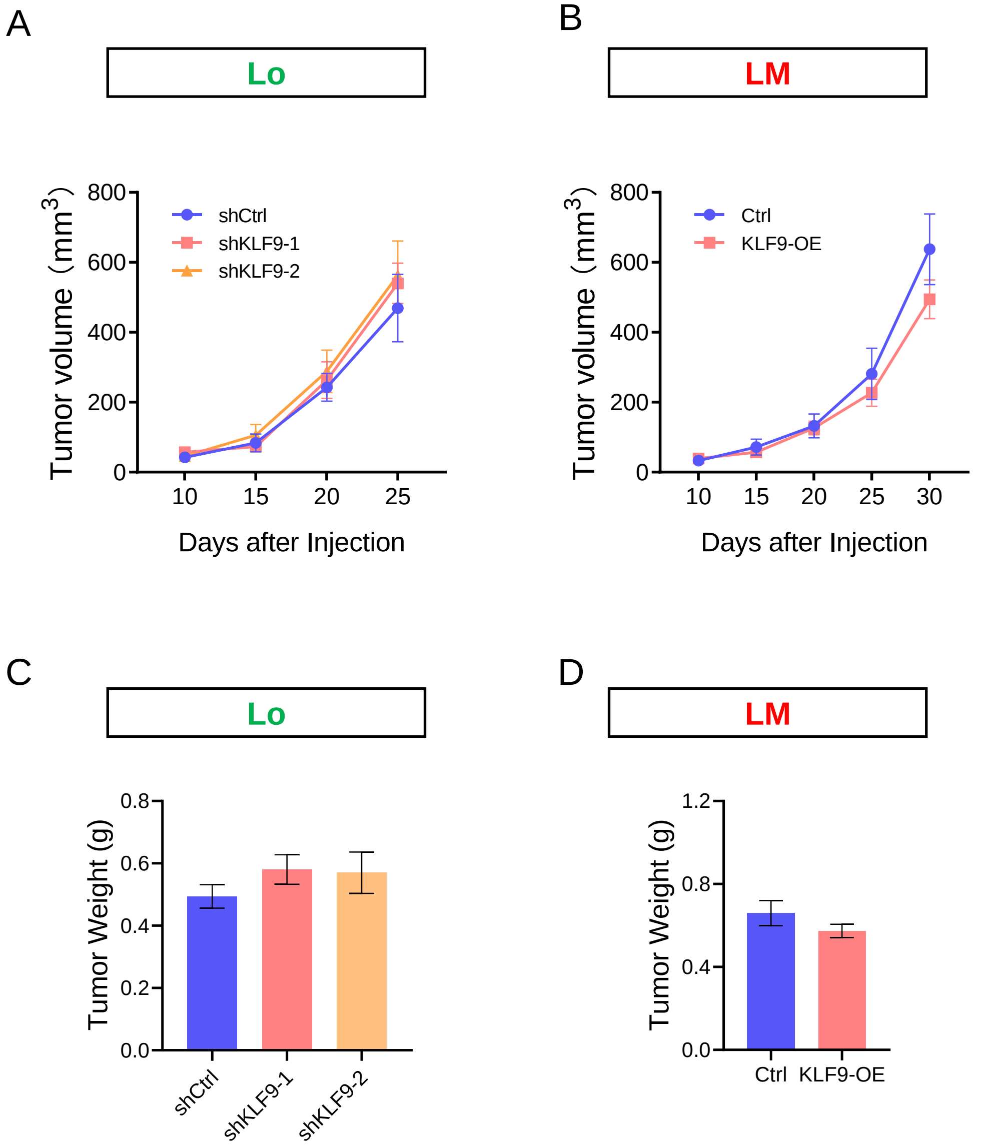

Fig.S5 KLF9 binds to the genomic locus of mesenchymal genes

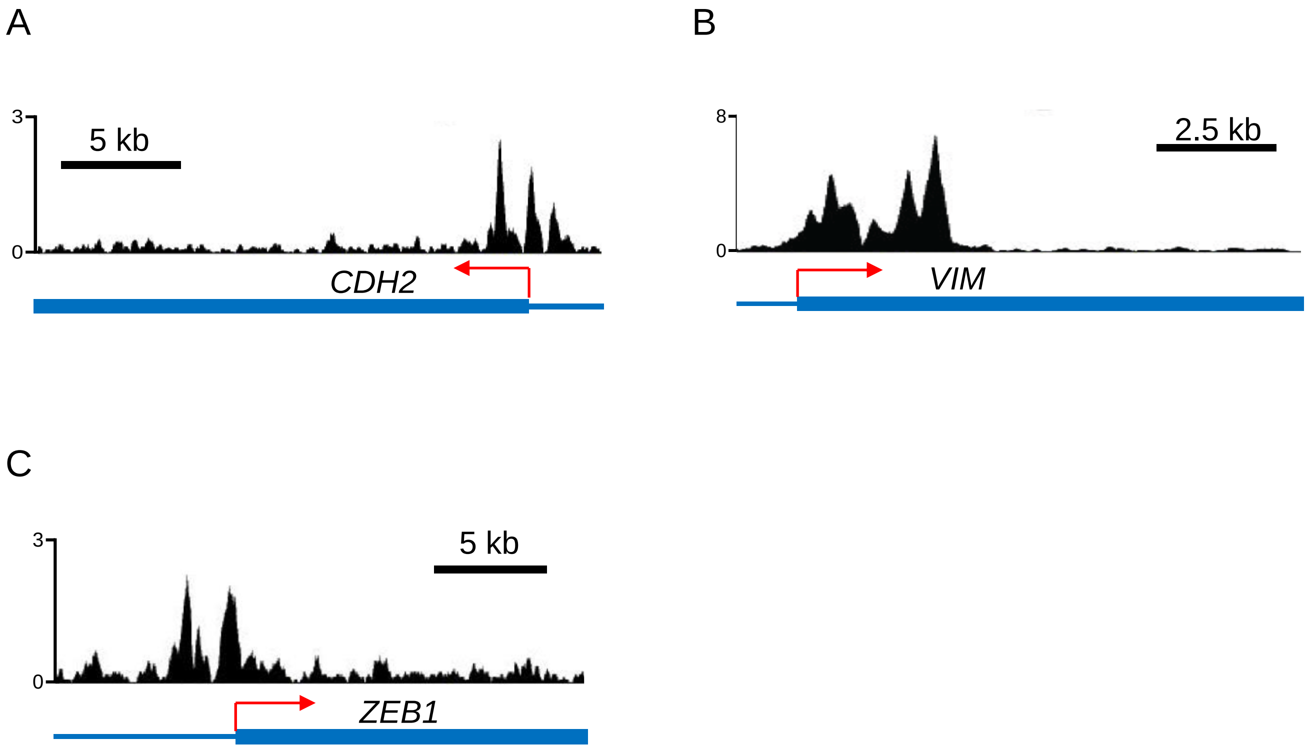

Supplement: Supplementary file 1 — Figure S1–S5 [file JCMM-27-2372-s001.pdf]
